# A Case of Myeloid Sarcoma following Allogeneic HSCT Presenting as Localized Hip Pain

**DOI:** 10.1155/2019/2674359

**Published:** 2019-10-02

**Authors:** Shannon Zhang, Casey Charlton, Akshay Amaraneni, Keri Maher

**Affiliations:** University of Arizona, Tucson College of Medicine, Tucson, AZ, USA

## Abstract

Myeloid sarcoma is a rare variant of acute myeloid leukemia (AML) which presents as an extramedullary soft tissue mass. Due to the rarity of this disease, along with nonspecific presenting symptoms, diagnosis can be delayed or missed without a high index of suspicion. In this case, we discuss a patient diagnosed with AML relapse in the form of myeloid sarcoma two years after allogeneic hematopoietic stem cell transplant (alloHSCT) for myelodysplastic syndrome (MDS) with the initial presentation for back pain misdiagnosed as spinal stenosis. This case report aims to help healthcare providers in recognizing the early signs and symptoms of this disorder as well as provide information in regards to treatment options and risk assessment.

## 1. Introduction

Myeloid sarcoma is a rare disease that can present as initial diagnosis or relapse of other hematologic disorders, most commonly acute myeloid leukemia (AML), as an extramedullary soft tissue tumor [1]. Due to the extremely low prevalence of this presentation, prospective knowledge on prognosis and optimal therapy is limited [1]. Presently, the literature consists of small retrospective studies or case reports. However, prognosis of myeloid sarcoma is thought to have a poorer prognosis than AML alone.

Myeloid sarcoma can precede, occur with, or occur after the diagnosis of AML, MDS, and other myeloproliferative neoplasms [2]. It can represent relapse after a hematopoietic cellular transplant and can occur in any organ. There has been a recent rise in reports of patients diagnosed with myeloid sarcoma after allogeneic stem cell transplant [1]. However, incidence is rare, occurring in less than 1% of transplanted patients. This form of relapse has been described up to five years after alloHSCT. Cases occurring after alloHSCT may represent failure of a graft-versus-leukemia effect with relapse of original disease or as a new therapy-related myeloid neoplasm [3].

In this case report, we discuss the findings of a 59-year-old Caucasian man presenting with back pain and leg weakness, who was ultimately diagnosed with myeloid sarcoma two years after receiving an allogeneic hematopoietic stem cell transplant for MDS.

## 2. Case Presentation

Our patient is a 59-year-old Caucasian male who was diagnosed in 2016 with MDS when he presented with thrombocytopenia (platelets 78,000) and neutropenia (ANC 800). Bone marrow biopsy showed trilineage dysplasia, and cytogenetics revealed a complex karyotype including −0, +3, 5q−, −7, +8, −9, and +22. He attained CR1 after 6 cycles of azacitidine, including normalization of cytogenetic studies. He underwent a reduced intensity matched unrelated donor peripheral blood stem cell transplant with fludarabine/melphalan + rabbit antithymocyte globulin conditioning in October 2016. His course was relatively uncomplicated, and one-year milestone visit revealed complete donor chimerisms and continued complete remission.

In March 2018, he began to complain of increasing pain in his lower back radiating down his right leg that ultimately resulted in decreased sensation and strength in his right leg. He was seen by a chiropractor, and also an orthopedic surgeon, and was diagnosed with spinal stenosis. His orthopedic surgeon planned on operating to relieve this stenosis, and preop labs showed new-onset thrombocytopenia for which he was referred back to our transplant center. Workup at the transplant center showed parvovirus and EBV positivity as well as worsening cytopenias. He was started on IVIG for treatment of parvovirus; however, this was ineffective. Interestingly, peripheral blood chimerisms at this time showed continued complete engraftment, and bone marrow biopsy morphology showed no evidence of relapsed MDS or AML and was read as a hypocellular marrow consistent with parvovirus infection.

**Figure 1:**
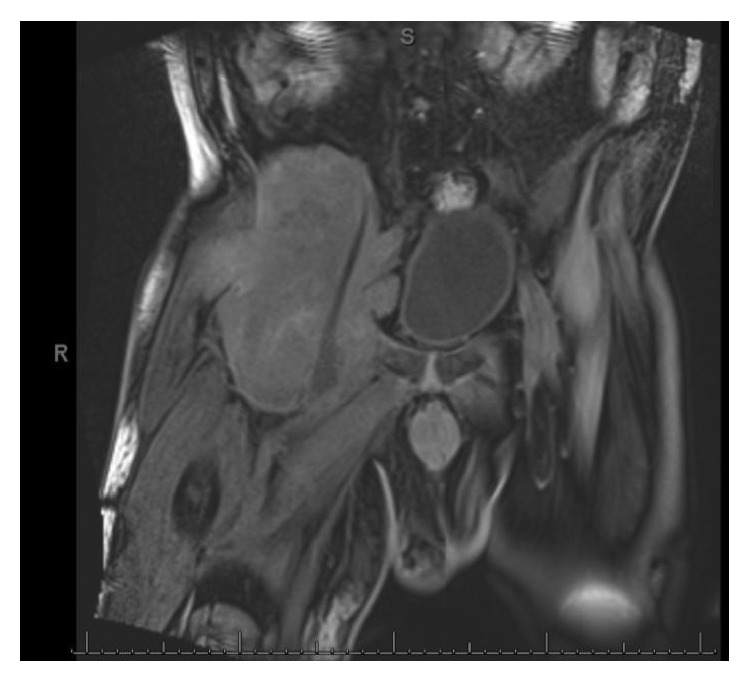
Large complex enhancing mass in the R iliac bone.

**Figure 2:**
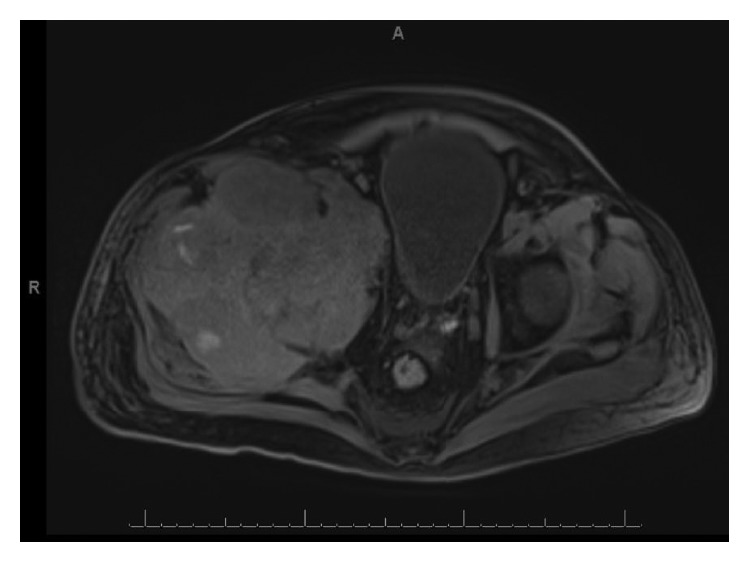
Large right iliac mass and associated destruction of surrounding osseous structures with involvement of right hip musculature.

A repeat MRI was performed due to worsening pain that detected a large right-sided pelvic mass in the right iliac bone with acetabular and femoral head destruction measuring 14.2 × 13.6 × 18.3 cm (Figures 1 and 2). This mass was biopsied, and the final pathology report indicated myeloid sarcoma (CD45+, CD34+, CD1117+, and MPO+). Ultimately, bone marrow cytogenetics which resulted nine days after his biopsy revealed complex karyotype, albeit with different findings from his initial diagnostic marrow, likely indicating clonal evolution of his original disease. Repeat peripheral blood chimerisms were performed on the blood two weeks after the initial presentation to our center, revealed rapidly declining donor chimerisms at 23% donor DNA.

Given the patient's poor functional status, he was not an intensive therapy candidate. After discussion of therapy options including palliative radiation, hospice, and palliative chemotherapy, he opted for palliative radiation. However, he continued to decline clinically and was discharged to hospice.

## 3. Discussion

Extramedullary leukemia, including myeloid sarcoma, occurs when myeloid cells form tumors outside the bone marrow. This may indicate dysfunctional homing signals for leukemic blasts via chemokines, metalloproteinases, and leukocyte surface beta2 integrins [4]. The type of myeloid sarcoma is categorized according to the most abundant cell type and whether the cells present are immature or mature. The tumor can be further phenotyped immunologically which may guide therapy options [5].

A study published in 2007, analyzed the clinical, cytogenetic, and phenotypic factors of 92 adult patients with myeloid sarcoma. The average age of those affected was 55.8 years ranging from 16 to 87 years with a male to female ratio of 1.42 : 1. In 28.2% of cases, myeloid sarcoma was found localized to the skin. Other common sites included the lymph nodes (16.3%), testes (6.5%), bone (3.25%), and CNS (3.25%) [6]. Twenty-seven percent of cases presented as de novo myeloid sarcoma with approximately 40% of these originally misdiagnosed most commonly as diffuse large B-cell lymphoma. Approximately, 35% presented with simultaneous AML, MPN, or MDS. The remaining 38% of individuals in the study had a previous history of AML, MPN, and MDS. Of those with a previous history of AML (12 patients), 42% were found to have the M2 subtype. Of those with a previous history of MPN (13 patients), CML was the most common cause with 54% being affected. Other causes of MPN present before a diagnosis of myeloid sarcoma included polycythemia vera, essential thrombocytopenia, and mastocytosis. Histological studies of all 92 cases in this study concluded that the most prevalent histotype was blastic (*N* = 42), followed by monoblastic (*N* = 20) and myelomonocytic (*N* = 20). Of the 92 cases retrospectively presented in this study, only seven were still living, six of whom received an allogeneic HSCT as part of their therapy. There were no differences in survival between the group with de novo myeloid sarcoma, concomitant hematological disease and myeloid sarcoma, or previous history of hematological disease. The mean survival of those who underwent chemotherapy, imatinib mesylate (for CML patients in the blast type), surgery, and radiation therapy was 7.1 months, 5.6 months, 36 days, and 1 week, respectively [6].

In AML myeloid sarcoma, therapy mimics that for medullary AML and follows the paradigm of intensive induction and consolidation, followed by allogeneic hematopoietic stem cell transplant in individuals who are candidates based on disease characteristics as listed in ELN 2017 and performance status/medical comorbidities. If alloHSCT is used as part of the therapy paradigm, those with complete donor chimerisms have improved overall survival compared to those with mixed chimerisms, as expected [7]. Palliative therapy options include modest dose radiation (20–30 Gy), azacitidine, supportive care, and hospice.

## 4. Conclusion

In conclusion, the patient discussed initially developed an isolated myeloid sarcoma two years after alloHSCT for MDS. His diagnosis was delayed by several weeks due to nonspecific presenting symptom of back pain and was only referred to hematology for further evaluation when he was incidentally noted to have thrombocytopenia on presurgical evaluation. At this time, the patient had declined significantly in functional status and was no longer a candidate for intensive therapy. While disease relapse after alloHSCT already portends a grim prognosis, this delay in diagnosis may have further worsened his overall prognosis. Vigilance for this diagnosis, especially in the post-transplant setting, is necessary due to its propensity for uncommon presentations.
